# Case Report: I want more olanzapine: pharmacogenetic insights into a patient's preference for high-dose olanzapine

**DOI:** 10.3389/fpsyt.2025.1633198

**Published:** 2025-07-10

**Authors:** Liam Korošec Hudnik, Ivo Kosmačin, Tanja Blagus, Vita Dolžan, Jurij Bon, Milica Pjevac

**Affiliations:** ^1^ Centre for Clinical Psychiatry, University Psychiatric Clinic Ljubljana, Ljubljana, Slovenia; ^2^ Pharmacogenetics Laboratory, Institute of Biochemistry and Molecular Genetics, Faculty of Medicine, University of Ljubljana, Ljubljana, Slovenia; ^3^ Department of Psychiatry, Faculty of Medicine, University of Ljubljana, Ljubljana, Slovenia

**Keywords:** case report, CYP1A2, CYP2D6, genetic polymorphism, olanzapine, personalized psychopharmacotherapy, pharmacogenetics, treatment resistance

## Abstract

Olanzapine is an effective antipsychotic agent, but its metabolism shows considerable interindividual variability. We present a case of a patient with treatment-resistant schizophrenia who consistently required and preferred high-dose olanzapine (40–60 mg/day) for symptom control. The patient reported improved motivation and energy following the reduction of adjunctive antipsychotics.

**Methods:**

The patient’s clinical course and treatment history were retrospectively reviewed. Plasma olanzapine levels were measured to assess systemic drug exposure, and pharmacogenetic testing for CYP1A2, CYP2D6, CYP3A4, and CYP3A5 polymorphisms was performed using PCR-based genotyping.

**Results:**

Genotyping revealed CYP1A2 -163AA genotype, consistent with an ultrarapid metabolizer phenotype, and CYP2D6 *1/*9 genotype, indicating slightly reduced but overall normal enzyme activity. At 40 mg/day, the olanzapine trough level was 51 ng/mL—lower than expected for a non-smoker—suggesting enhanced metabolic clearance. This pharmacokinetic profile, shaped by genetic predisposition and smoking, likely necessitated higher olanzapine doses to reach therapeutic levels. Discontinuation of haloperidol and risperidone was associated with improved subjective energy and engagement.

**Conclusion:**

This case illustrates how pharmacogenetic variability may influence antipsychotic efficacy and tolerability. The patient’s ultrarapid CYP1A2 metabolism and smoking status likely reduced olanzapine exposure, warranting higher doses for clinical response. Pharmacogenetic profiling may provide valuable insights into individual treatment needs and support more personalized approaches in complex psychiatric cases.

## Introduction

Olanzapine is one of the most extensively studied second-generation antipsychotics due to its commonly perceived efficacy, good tolerability, and significant metabolic side effects. In phase 1 of the CATIE schizophrenia study, olanzapine demonstrated superior outcomes on primary efficacy measures compared to the other second-generation antipsychotics and perphenazine (1). It is however important to note that the primary outcome measure in question was “all-cause discontinuation”, therefore the apparent superiority of olanzapine may, in part, be attributed to its high subjective tolerability. Due to its strong binding affinity for serotonergic 5-hydroxytryptamine 2A receptor (5-HT_2A_) receptors, olanzapine is associated with a lower incidence of subjectively distressing adverse effects, such as parkinsonism, akathisia, and other dysphoric experiences linked to excessive dopaminergic type 2 (D2) receptor blockade (2, 3). Some authors have suggested that olanzapine’s efficacy may rival that of clozapine among patients with treatment-resistant schizophrenia (TRS), but this may represent an overestimation due to differences in inclusion criteria across studies. Although olanzapine has not consistently been shown to match the efficacy of clozapine when rigorous TRS criteria are applied, it may offer benefits for a specific subgroup of therapeutically resistant patients. Gannon and colleagues reviewed studies evaluating the efficacy, safety, and tolerability of high-dose olanzapine. Based on their analysis of 10 studies, they concluded that olanzapine doses exceeding 20 mg/day may be more effective than haloperidol and commonly used second-generation antipsychotics in TRS cases where clozapine is either intolerable or contraindicated (4).

Olanzapine is primarily metabolized by the cytochrome P450 enzyme CYP1A2 or via direct glucuronidation. Because of enzyme induction, smokers tend to have lower serum concentrations and higher clearance rates of olanzapine compared to nonsmokers. The therapeutic effects are attributed to olanzapine, while its metabolism mainly serves as a pathway for inactivation and elimination. The resulting metabolites,10-N-glucuronide and 4N-glucuronides, are considered pharmacologically inactive or, in the case of 4-Ndesmethylolanzapine, minimally active but clinically insignificant. In addition to tobacco use, sex also has a significant impact on metabolism. However, there is not sufficient data supporting the necessity for the dose adjustment in individuals with faster metabolism. In contrast, there is some evidence that lower doses are required in individuals with slower metabolism—such as women, older adults, and non-smokers (5, 6). Olanzapine serum concentrations show strong correlation with cerebrospinal fluid levels and can be readily measured; therefore, therapeutic drug monitoring is a valuable tool for optimizing treatment efficacy and ensuring patient safety (7). Several studies with differing methodologies show a high degree of agreement on the plasma level response threshold for olanzapine at 23 ng/ml (8–10).

In this case report, we present a patient with TRS and poor tolerability to clozapine, who was consequently treated with a combination of antipsychotics with only partial effectiveness, until a high dose of olanzapine led to satisfactory remission. Particularly notable was the patient’s consistent personal preference for higher doses of olanzapine. Pharmacogenetic testing of CYP1A2 and CYP2D6 revealed specific genetically determined metabolic characteristic, which may potentially explain the good response and the expressed preference for high daily olanzapine dose, the partial efficacy and side effects observed with clozapine, as well as patient’s reluctance to risperidone and haloperidol.

## Case description

A 36-year-old male patient with a diagnosis of schizophrenia, previously treated at another facility, first attended our outpatient unit in June 2022. He did not have any other medical conditions requiring treatment up until then and had no family history of psychiatric disorders. He was a smoker, smoking up to one pack of cigarettes a day, but denied consuming alcohol or prohibited psychoactive substances, although he disclosed smoking marihuana occasionally in his youth.

We reconstructed the patient’s preceding treatment history based on the available medical records and the patient’s own account. He was first admitted to an intensive psychiatric care unit at the age of 20 years in 2006. Due to psychotic symptoms, he was prescribed olanzapine 5 mg and lorazepam 1 mg QHS. He was discharged after four days, at his own request and against medical advice, but in accordance with the absence of legal criteria for involuntary treatment. He discontinued the prescribed antipsychotic medication. In March 2009 he sought help in the psychiatric emergency unit due to intense fear and anxiety. He presented with persecutory ideations. His friends and family described that he reported on acoustic hallucinations, passivity experiences, and telepathy. According to collateral reports, he had become progressively withdrawn and inactive, and his relationship with his parents had become increasingly strained. He was voluntarily admitted to in-hospital treatment at the intensive psychiatric ward of the University Psychiatric Clinic Ljubljana. A diagnostic work-up following the protocol for first-episode psychosis included a physical and neurological examination, routine blood tests (including complete blood count, liver and kidney function tests, thyroid function, and inflammatory markers) and serological testing for infectious and autoimmune causes (e.g., HIV, syphilis, and antinuclear antibodies). The results of all aforementioned tests were normal, a head CT revealed no structural abnormalities, and an electroencephalogram (EEG) was also performed to exclude seizure activity. Treatment with amisulpride up to 600 mg/day TID and later olanzapine up to 20 mg/day QHS was initiated. Due to an inadequate response to consecutive trials of two antipsychotic medications and the persistence of psychotic symptoms, treatment with clozapine was initiated. Olanzapine was discontinued, and clozapine was slowly titrated alongside amisulpride 400 mg/day BID up to 500 mg/day BID. Following the introduction of clozapine, the patient’s condition gradually improved with a significant reduction in positive symptoms. He was discharged with a recommendation to continue treatment in the outpatient setting at a facility in another location, which was more suitable at the time, given his living arrangements.

Between 2009 and 2019, he attended regular check-ups, at first once a month, later once every two months. Amisulpride was gradually discontinued with no recorded deterioration in his mental state. Due to continuous complaint of excessive daytime sedation and hypersalivation the dose of clozapine was slowly tapered to 350 mg/day QHS. Despite the lower dosage, the patient frequently reported hypersalivation and expressed a desire to discontinue the treatment. At the same time, he often complained of insomnia, for which his treating psychiatrist prescribed zolpidem 5 mg as needed at bedtime.

In October 2019, he was hospitalized for the third time at the intensive psychiatric ward of the University Psychiatric Clinic Ljubljana due to a deterioration in his mental state following complete discontinuation of treatment. After an incident of aggression in his home environment, he was admitted involuntarily. He exhibited acute psychotic symptoms and psychomotor agitation. Upon admission, treatment with risperidone and lorazepam was initiated to manage agitation; later clozapine was reintroduced. In the third week of hospitalization, the patient developed a high fever, reaching 38.8°C. As a precaution, clozapine—at that time administered at a dose of 75 mg/day—was temporarily discontinued. The elevated body temperature was attributed to a localized inflammatory process in the right mandibular region, which required surgical drainage and a course of antibiotic therapy. Following completion of the antibiotic treatment, the patient became reluctant to resume clozapine due to previously experienced adverse effects, particularly severe hypersalivation. Given the need to address residual psychotic symptoms and the patient’s unwillingness to resume previously effective clozapine treatment, the attending psychiatrist adopted a polypharmacological approach. His condition gradually improved, and he was discharged with olanzapine 20 mg/day QHS, risperidone 8 mg/day BID, haloperidol 4 mg/day TID, and clonazepam 3 mg/day TID.

Following his discharge in December 2019 and until his enrollment in our outpatient service, the patient continued outpatient treatment under the care of his previous psychiatrist. He attended monthly check-ups, during which clonazepam was gradually tapered and eventually discontinued. Good control of positive psychotic symptoms and adherence to the prescribed treatment were documented. However, the patient continued to report excessive daytime sedation alongside persistent sleep difficulties, for which he was occasionally prescribed zolpidem at a dose of 5 mg. Due to persistent complaints of insomnia after unsuccessful attempts with non-pharmacological interventions as well as trazodone, the treating psychiatrist increased the olanzapine dose to 30 mg/day, after which the patient reported an improvement in sleep. Weight gain was observed during the course of the prescribed treatment, and the patient was referred to a weight management program organized by the local primary health care center.

The patient expressed a preference to continue treatment at our outpatient unit, citing its proximity to his place of residence as a matter of convenience. He presented for an initial consultation in June 2022. At this time, he was prescribed olanzapine 30 mg/day QHS, risperidone 8mg/day, haloperidol 4mg/day TID, and trazodone 50 mg in the evening. The latter was ineffective in treating his persistent insomnia, and he admitted to having discontinued it. He was free of acute psychotic symptoms and demonstrated insight into the nature of his condition and the need for maintenance antipsychotic therapy. He again emphasized experiencing severe and persistent sleep disturbances. In addition, he reported excessive daytime sleepiness and a lack of motivation. He was overweight with a BMI of 36, had normal ECG without QTc prolongation, and presented with no clear extrapyramidal side effects of medication. Routine blood tests revealed mixed dyslipidemia, for which rosuvastatin was subsequently initiated. Given the patient’s polypharmacy and established metabolic syndrome, we proposed a gradual adjustment of his pharmacotherapy to transition to a metabolically more favorable monotherapy. At first the dose of olanzapine was gradually tapered at a rate of 5 mg per month. After two months, at a dose of 20 mg/day, the patient voiced his objection to further dose reduction. He expressed a preference to continue olanzapine while discontinuing other medications. He reported that olanzapine was the most effective treatment for his symptoms, caused the least muscular tension, and was the only medication that alleviated his insomnia. In the period between follow-up appointments, the patient frequently contacted the outpatient clinic, repeatedly requesting additional prescriptions specifically for olanzapine, often citing various reasons such as having lost his medication. Upon confrontation, the patient admitted to taking olanzapine at a higher dose than prescribed as he found it helpful for sleep. He also disclosed that, on rare occasions when unable to fall asleep, he took up to 60 mg. He reported adhering to the prescribed regimen for the other medications but felt they were ineffective. He experienced fatigue and lack of motivation following the morning doses. The patient was thoroughly educated about the negative impact of olanzapine on metabolic health. Nevertheless, he persisted in his preference for olanzapine, explicitly stating, ‘If it’s acceptable, I would like to continue taking 40 mg of olanzapine, as it is the only thing that helps me sleep.’ Taking into account the patient’s preferences and to maintain adherence to maintenance treatment, we agreed to prescribe olanzapine at a dose of 40 mg/day QHS. Concurrently, a plan was established for the gradual tapering of the other two antipsychotic medications. The patient was encouraged to adopt a healthy, balanced diet and engage in regular physical activity with the goal of weight reduction.

As a safety measure and to objectively assess treatment adherence, a trough plasma level of olanzapine was measured using liquid chromatography–mass spectrometry (LC-MS). A venous blood sample was obtained 12 hours after the patient’s last evening dose of olanzapine. The analysis revealed a plasma olanzapine concentration of 51 ng/mL. The patient reported a consistent daily intake of 40 mg of olanzapine in the evening. The dose of haloperidol was gradually tapered until discontinuation, and the dose of risperidone was also reduced. Following the discontinuation of haloperidol, and while receiving olanzapine 40 mg/day QHS and risperidone 6 mg/day TID, the patient spontaneously reported experiencing increased motivation and energy throughout the day. He reported improved ability to engage in physical activity. No worsening of psychotic symptoms was observed during this period.

**Figure 1:**
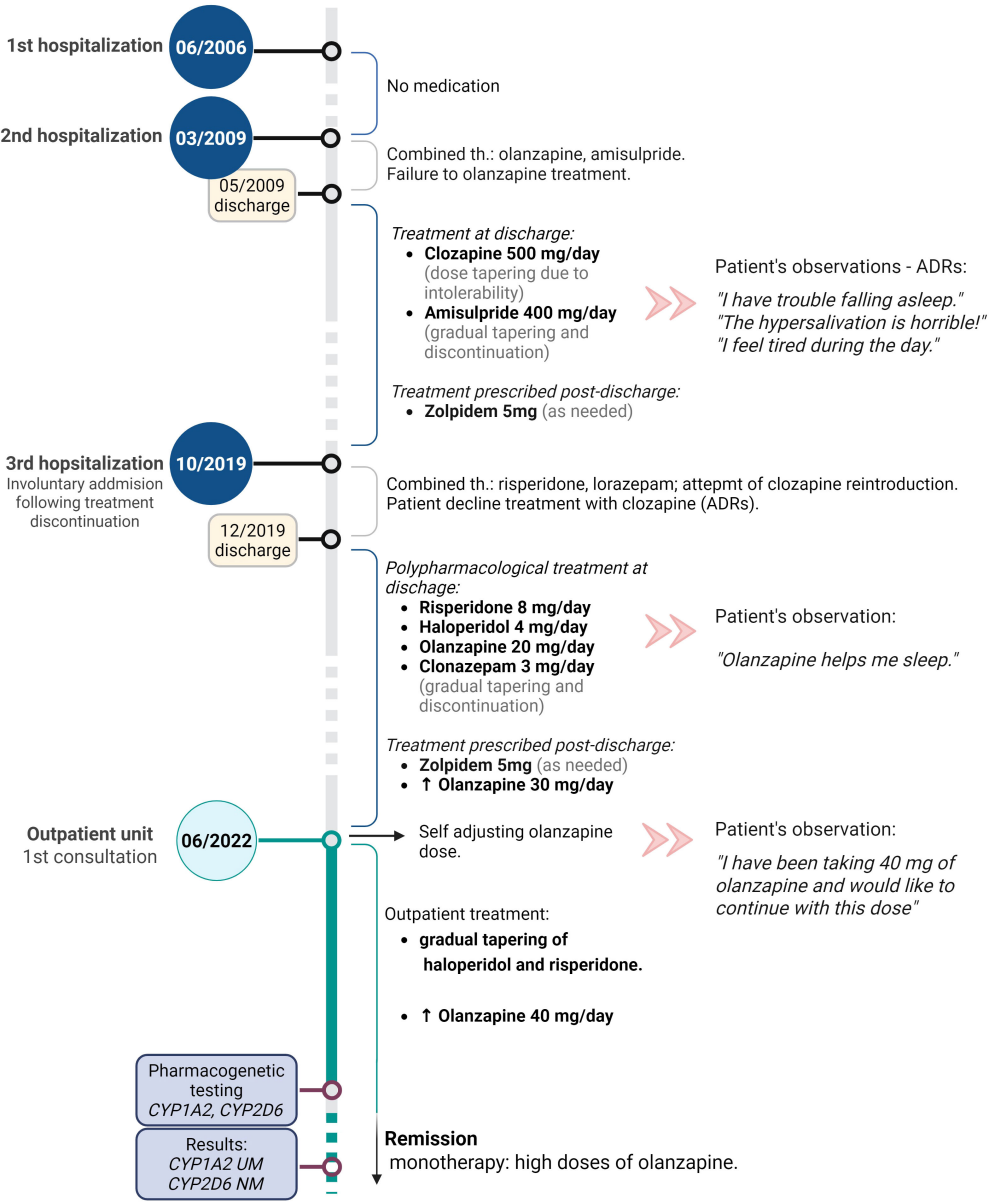
Graphical representation of the patient’s treatment timeline, including antipsychotic dosing, medication changes, and key clinical events.

To elucidate potential pharmacogenetic factors underlying the patient’s atypical therapeutic response and preference for higher olanzapine doses, a targeted analysis of common functional polymorphisms in the CYP1A2, CYP2D6, CYP3A4, and CYP3A5 was conducted after obtaining informed consent. A graphical representation of the patient’s treatment history is provided in **Figure 1**.

## Methods

Pharmacogenetic testing was performed using DNA extracted from peripheral blood leukocytes with the E.Z.N.A. SQ Blood Kit II (Omega Bio-tek), following the manufacturer’s protocol. Copy number variations in CYP2D6, including gene deletions (CYP2D6*5) and duplications (CYP2D6*xN), were assessed using long-range PCR (Biotechrabbit GmbH) and confirmed by gel electrophoresis.

Single-nucleotide variants (SNVs) in CYP1A2 (rs762551, *30) and CYP2D6 (including common variants such as *3, *4, *6, *9, *10, *17, *41) were analyzed using KASP genotyping assays (LGC Biosearch Technologies), which detect allele-specific PCR products via fluorescence. All assays were performed in duplicate with appropriate positive and negative controls.

Pharmacogenetic testing was performed using DNA extracted from peripheral blood leukocytes with the E.Z.N.A. SQ Blood Kit II (Omega Bio-tek, USA), following the manufacturer’s instructions. Copy number variations in CYP2D6, including gene deletion (CYP2D6*5) and duplication (CYP2D6*xN), were assessed using long-range PCR

(Biotechrabbit GmbH, GER) and confirmed by gel electrophoresis. Single-nucleotide variants

(SNVs) in CYP1A2 (-163C>A (rs762551)), CYP2D6 (*3, *4, *6, *9, *10, 14A/B, *17, *41),

CYP3A4 (*22) and CYP3A5 (*3, *6, *7) were analyzed using KASP genotyping assays (LGC Biosearch Technologies, UK). All analyses were performed in duplicate along with appropriate controls.

## Results and discussion

Summary of the genotyping analysis and the patient’s phenotypes is presented in **Table 1**. Considering the genotypes and resulting phenotypes, we attempted to retrospectively elucidate the drugs’ pharmacokinetics to explain the patient’s treatment response and preference for high-dose olanzapine, although evidence-based recommendations for treatment selection in such cases are not yet established.

**Table 1:** Summary of the genotyping analysis and the patient’s phenotypes.

Gene	Analyzed polymorphisms	Genotype	Phenotype
CYP1A2	-163C>A	-163AA	UM (ultrarapid metabolizer)
CYP2D6	*3, *4, *5, *6, *8, *9, *10, *14A/B, *17, *41, xN	*1/*9	NM (normal metabolizer)
CYP3A4	*22	*1/*1	NM (normal metabolizer)
CYP3A5	*3, *6, *7	*3/*3	PM (poor metabolizer)

The presence of the CYP1A2 -163AA genotype indicates homozygosity for a genetic variant in the promoter region of the CYP1A2 gene that increases inducibility and thereby gene expression. This could lead to accelerated metabolism of certain drugs when coadministered with the inducers of this enzyme. Consequently, the efficacy of certain antipsychotics that are primarily (e.g., olanzapine, asenapine, thiothixene, trifluoperazine) or partially (e.g., clozapine) metabolized through CYP1A2 (11). Numerous studies suggest that CYP1A2 activity is more strongly influenced by CYP1A2 expression levels than by genotype alone (12, 13). Polycyclic aromatic hydrocarbons present in tobacco smoke are common inducers of CYP1A2. Smokers have lower plasma concentrations of olanzapine compared to non-smokers, with a reported mean decrease in trough levels of up to 35–45% (14). Increased olanzapine clearance due to CYP1A2 induction lowers exposure (area under the curve) and shortens drug half-life. Subtherapeutic levels may occur in smokers at standard olanzapine doses (e.g., 10 mg/day). This may lead to reduced efficacy, increased risk of relapse, or the need for higher doses, although the clinical relevance and need for dose adjustment are disputed, and no clear evidence-based treatment recommendations are available.

**Figure 2:**
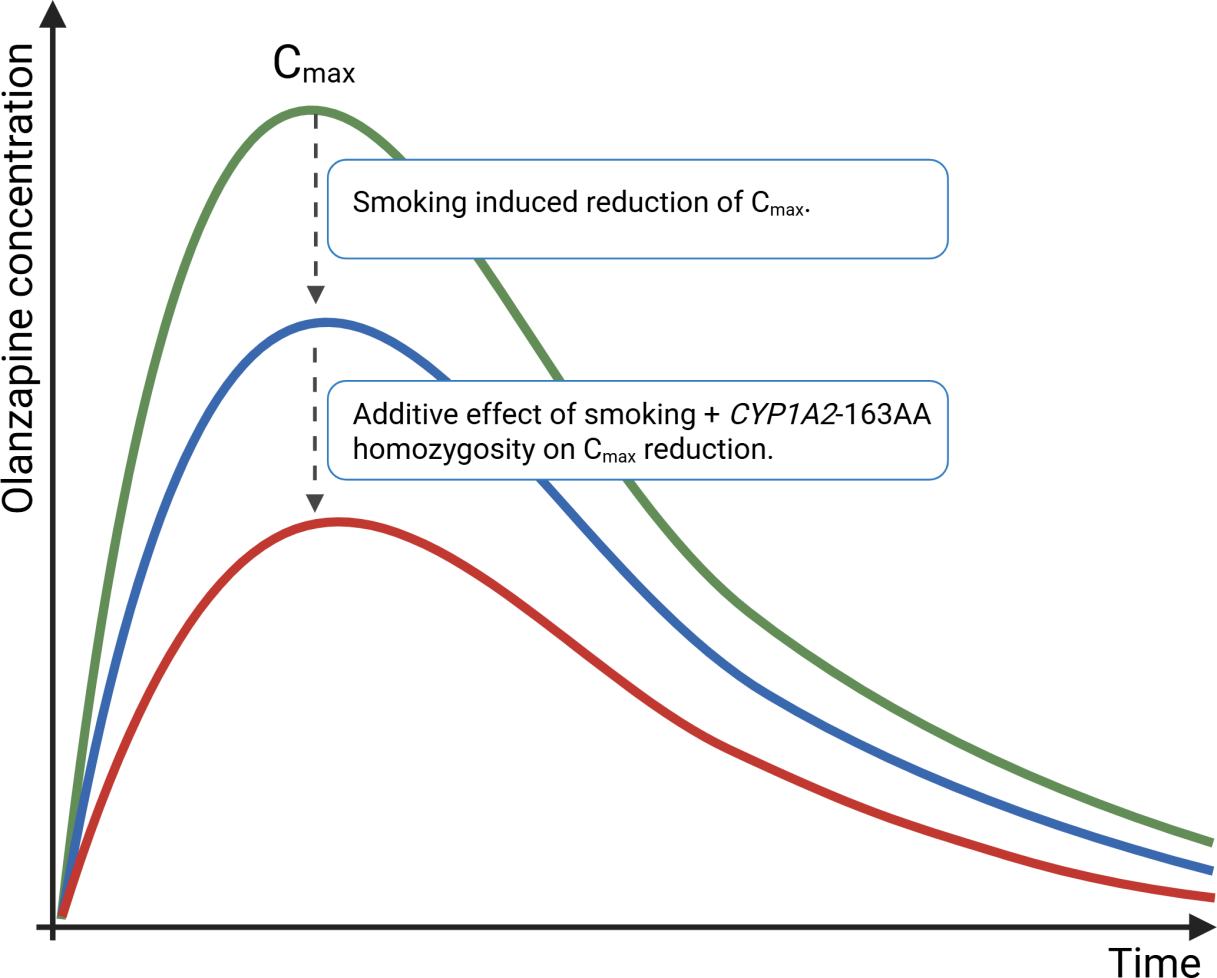
Schematic representation of the proposed impact of smoking and CYP1A2 phenotype on olanzapine pharmacokinetics, illustrating reduced plasma concentrations and altered peak–trough dynamics. The schematic is based on published data describing olanzapine pharmacokinetics and the known effects of smoking and specific CYP1A2 metabolic phenotypes on drug metabolism (5, 14, 15).

The effect of smoking on enzyme induction is theoretically more pronounced in individuals with the CYP1A2 -163AA genotype, which is associated with the phenotype of ultrarapid metabolizer (UM) (15). In the case of the patient presented, we hypothesize that accelerated olanzapine metabolism contributed to a significantly reduced concentration-to-dose ratio. This pharmacokinetic profile may explain the patient’s subjective preference for higher doses. The administration of elevated doses (40–60 mg) likely compensated for enhanced metabolic clearance by achieving higher peak plasma concentrations shortly after ingestion. This, in turn, may account for the patient’s reported sedative effects only at higher doses, as supported by olanzapine’s concentration-time kinetics. These observations are consistent with the known pharmacological characteristics of olanzapine, wherein peak plasma levels play a critical role in mediating sedative and anxiolytic effects (5). The proposed cumulative effects on olanzapine pharmacokinetics are illustrated schematically in **Figure 2**.

One limitation of this study is the absence of direct assessment of CYP1A2 expression, which would have provided definitive evidence of enhanced enzyme induction. However, CYP1A2 activity was indirectly inferred through measured olanzapine plasma concentrations and the patient’s clinical response. Plasma olanzapine levels were measured following the initiation of high-dose olanzapine, primarily to objectively assess adherence, as the patient’s persistent request for higher doses was atypical compared to standard clinical experience. The measured trough plasma concentration of olanzapine was 51 ng/mL at a nightly dose of 40 mg (QHS). This value supports the hypothesis of accelerated olanzapine metabolism in this patient. Previous pharmacokinetic studies have demonstrated an approximate conversion factor of 2 between daily dose and expected 12-hour trough levels in nonsmokers, suggesting that a 40 mg daily dose would typically yield a trough concentration of approximately 80 ng/mL in the absence of metabolic inducers or individual metabolic variations (16). The observed discrepancy further supports the presence of enhanced olanzapine clearance, potentially due to a combination of smoking and genetic factors influencing CYP1A2 inducibility.

The patient developed features of metabolic syndrome during high-dose olanzapine treatment, raising safety concerns. However, due to his strong preference for continuing olanzapine and resistance to switching to a potentially more metabolically favorable antipsychotic, management focused on lifestyle modifications and the initiation of statin therapy to address hyperlipidemia. No other clear adverse drug reactions (ADR) were observed. The measured trough plasma concentration of 51 ng/mL exceeds the previously proposed therapeutic response threshold of approximately 20 ng/mL for the majority of patients (17). On the other hand, concerning safety and tolerability, previous studies have demonstrated that high-dose olanzapine treatment—and even trough plasma concentrations exceeding 200 ng/mL—can be well tolerated by a substantial subset of patients (18, 19).

Furthermore, the patient’s CYP1A2 metabolic phenotype likely contributed to a suboptimal therapeutic response to clozapine. Increased CYP1A2 activity can lead to faster clearance of clozapine, resulting in lower plasma concentrations and reduced clinical efficacy. While CYP3A4 and CYP3A5 enzymes also influence clozapine metabolism—particularly its conversion to norclozapine— (20, 21) they were likely less influential in this case. The patient was a normal metabolizer for CYP3A4 and a poor metabolizer for CYP3A5, suggesting minimal impact on overall clozapine clearance from these pathways. The increased production of norclozapine, clozapine’s major metabolite, may have affected the side effect profile. Norclozapine has weaker antipsychotic properties, a longer half-life, and is associated with hypersalivation (sialorrhea) (22, 23). Although plasma levels were not measured during clozapine treatment, the patient’s report of severe hypersalivation— ultimately contributing to his refusal of clozapine reintroduction during his third hospitalization—is consistent with this proposed mechanism.

The patient also carried CYP2D6 *1/*9 genotype which is according to current guidelines associated with the normal metabolizer (NM) phenotype, albeit with slightly reduced enzymatic activity (activity score 1.5) (24). Nevertheless, the slightly reduced enzymatic activity may still result in slower conversion of risperidone to its active metabolite, paliperidone. This may lead to higher plasma concentrations of risperidone relative to paliperidone, without significantly affecting the total active moiety. In individuals with reduced CYP2D6 enzymatic activity, this could result in disproportionately higher risperidone levels, potentially leading to increased D2 receptor occupancy at a given daily dose. Haloperidol is primarily metabolized by CYP3A4 and, to a lesser extent also CYP2D6. We excluded the potential impact of CYP3A4 and CYP3A5 genotypes on haloperidol metabolism, as the patient was a normal CYP3A4 and poor CYP3A5 metabolizer. However, the patient’s mildly reduced CYP2D6 enzymatic activity may have influenced plasma concentrations of haloperidol. All above-described mechanisms could hypothetically have led to enhanced D2 receptor blockade during the period of polypharmacological treatment when haloperidol and risperidone were co-administered in the presented patient. Although no extrapyramidal symptoms were described, excessive D2 receptor blockade can exacerbate negative and cognitive symptoms and attenuate reward-related processing leading to impairments in motivated behavior, hedonic experience, and emotional expression (25). Although not assessed using objective measures, the patient’s reported increase in motivation and energy throughout the day, along with improved capacity to engage in physical activity following the tapering of haloperidol and risperidone, may reflect a reduction in excessive D2 receptor blockade.

Apart from the proposed pharmacokinetic mechanisms that may hypothetically explain the patient’s preference for high-dose olanzapine, this case also warrants consideration of potential non-therapeutic use or misuse-like behavior involving olanzapine. Recent literature highlights a notable potential for misuse and abuse among certain atypical antipsychotics. Although no definitive mechanism of action underlying possible reinforcing effects has been clearly established, agents such as olanzapine and quetiapine may elicit non-therapeutic seeking behavior in some individuals, likely due to their pronounced sedative and anxiolytic properties (26). Unlike substances with well-established misuse potential, reports of olanzapine abuse in the literature remain relatively scarce (27). However, the present case may be alternatively interpreted through the lens of misuse and drug-seeking behavior commonly observed in substance use disorders.

## Conclusions

This case illustrates how pharmacogenetic and pharmacokinetic factors may underlie atypical treatment responses in psychiatry. The patient’s CYP1A2 -163AA genotype, combined with smoking, likely led to accelerated olanzapine metabolism, possibly explaining the need for higher doses to achieve therapeutic effects and contributing to clozapine inefficacy and hypersalivation. Additionally, the CYP2D6 *1/*9 genotype may have altered risperidone and haloperidol metabolism, potentially increasing D2 receptor blockade and dampening motivation and affect—effects that improved after discontinuation of these agents. Although routine genotype-guided prescribing is not yet supported by sufficient evidence, this case illustrates how pharmacogenetic testing may provide useful insights in selected treatment-resistant patients. Further research is needed to clarify its role in personalized psychopharmacology.

## Data Availability

The original contributions presented in the study are included in the article, further inquiries can be directed to the corresponding author/s.
